# JP3, an antiangiogenic peptide, inhibits growth and metastasis of gastric cancer through TRIM25/SP1/MMP2 axis

**DOI:** 10.1186/s13046-020-01617-8

**Published:** 2020-06-23

**Authors:** Jun-Jie Chen, Yan-Lin Ren, Chuan-Jun Shu, Yi Zhang, Min-Juan Chen, Jin Xu, Jin Li, Ai-Ping Li, Dong-Yin Chen, Jing-Dong He, Yong-Qian Shu, Jian-Wei Zhou

**Affiliations:** 1 Department of Molecular Cell Biology & Toxicology, Center for Global Health, School of Public Health, Nanjing Medical University, Nanjing, 211166 China; 2 Jiangsu Key Lab of Cancer Biomarkers, Prevention and Treatment, Collaborative Innovation Center for Cancer Medicine, Nanjing Medical University, Nanjing, 211166 China; 3 Department of Bioinformatics, School of Biomedical Engineering and Informatics, Nanjing Medical University, Nanjing, 211166 China; 4 Department of Oncology, the affiliated Huaian No. 1 People’s Hospital of Nanjing Medical University, Huaian, Jiangsu Province China; 5 Department of Medicinal Chemistry, School of Pharmacy, Nanjing Medical University, Nanjing, 211166 China; 6 Department of Oncology, The First Affiliated Hospital of Nanjing Medical University, Nanjing, 211166 China

**Keywords:** Targeting peptide, JP3, TRIM25, SP1, Ubiquitination, Gastric cancer, Angiogenesis, Experimental therapy

## Abstract

**Background:**

Gastric cancer (GC) is the most prevalent gastrointestinal tumor with an unfavorable clinical prognosis. GC patients are largely threatened owing to metastasis and drug resistance. Tumor angiogenesis plays an important role in the development of gastric cancer and is a challenge in the treatment of gastric cancer.

**Methods:**

Mouse xenograft models were used for screening of therapeutic peptides on GC growth and metastasis. Routine laboratory experimental methods including conditional cell culture, tube formation assay, qRT-PCR, Western blotting, immunohistochemistry (IHC), ubiquitination assay, and immunofluorescence (IF) were used in mechanism investigation; protein docking analysis and coimmunoprecipitation (Co-IP) were used for prediction and confirmation of interactions between JP3/SP1 and TRIM25/MEK1/2.

**Results:**

We identified an MMP2-targeted peptide JP3 that plays inhibiting roles in modulating growth and metastasis of GC in vivo and has no observable toxic side effects. JP3 reduced tumor microvessel density (MVD) in vivo and human umbilical vein endothelial cells (HUVECs) tube formation in vitro. Mechanistic studies revealed that JP3 reduces polyubiquitination-mediated degradation of TRIM25 by increasing the stability of TRIM25 through phosphorylating it at Ser12. TRIM25, as an E3 ubiquitin ligase, promoted the ubiquitin of SP1 at K610, further suppressed expression of MMP2 and inhibited angiogenesis in GC. Importantly, the inversely association between TRIM25 and SP1 protein level was further verified in human GC tissues. Decreased TRIM25 expression and increased SP1 expression in tumor tissues were positively correlated with poor prognosis of GC patients.

**Conclusions:**

MMP2-targeted peptide JP3 plays a therapeutic role in GC through anti-angiogenesis by modulating TRIM25/SP1/MMP2.

## Background

Gastric cancer (GC) is one of the most common gastrointestinal malignancies. It has the fifth highest global incidence and the third highest death rate in all malignancies [1, 2]. Although the survival of GC patients has improved with advances in diagnostic and therapeutic techniques, more than 90% of patients eventually are still died from metastasis [3]. GC grows in situ and spreads through blood circulation, leading to distant metastasis [4]. In this process, tumor angiogenesis exerts a crucial role. There is an urgent need for new therapeutic approaches especially in anti-angiogenesis.

Matrix metalloproteinases 2 (MMP2) is an important member of the matrix metalloproteinases family, which is of significance in the degradation of tumor-mediated extracellular matrix (ECM) [5]. Degradation of basement membrane and matrix is extremely important for endothelial cell migration, a necessary event involved in angiogenesis [6, 7]. Previous studies have proven that MMP2 overexpression promotes the proliferation, angiogenesis and migration of GC [8–10]. However, JWA down-regulates MMP2 by inhibition of transcription factor SP1, thus suppressing the angiogenesis of GC. In GC cells, on the hand, JWA is interacted with RNF185 and degraded by ubiquitin-proteasome pathway [11]; and on the other hand, increased JWA expression promotes the degradation of SP1 by ubiquitination related mechanism, however, the specific E3 ubiquitin ligase of SP1 is undetermined [12, 13]. Erkki Koivunen et al. isolated a novel class of peptides containing the histidine-tryptophan-glycine-phenylalanine (HWGF) motif which could specifically recognize MMP2 from the phage display libraries of random peptides [14]. The peptides containing the HWGF sequence were found to have 10 times more uptake in the vasculature of tumor tissues than control [15]. These previous studies suggested that HWGF could be used as a useful target peptide linked to other therapeutic peptide in experimental cancer therapy.

In this study, a number of candidate peptides based on the functional characteristics of JWA protein were designed and screened their anti-GC activities by subcutaneous xenograft nude mouse models. MMP2-targeted JP3 was finally determined to be the best anti-cancer peptide without detectable side effects in animal models, showing a therapeutic efficacy. Mechanistically, we identified that JP3 stabilized an E3 ubiquitin ligase TRIM25 by phosphorylation and delayed its degradation. TRIM25 further degraded SP1 therefore reduces transcription and translation of MMP2, ultimately leading to reduction of angiogenesis and tumor proliferation in GC. Our results may provide new insight to clinical targeted therapy of GC, and JP3 may benefit GC patients as a novel effective and safe anti-angiogenesis agent.

## Materials and methods

### Cell lines and culture

Human metastatic GC cell line BGC823 was gifted from Professor Q Guo, China Pharmaceutical University. The SGC7901 and MGC803 cell lines were obtained from the Type Culture Collection of the Chinese Academy of Sciences (Shanghai, China). Human umbilical vein endothelial cells (HUVECs) were purchased from the Shanghai Institute of Biochemistry and Cell Biology, Chinese Academy of Sciences (Shanghai, China). HUVECs, BGC823 and SGC7901 cells were cultured in RPMI-1640 (Hyclone, Thermo Scientific, Waltham, MA, USA), while MGC803 cells were maintained in Dulbecco’s Modified Eagle Medium (DMEM), supplemented with 10% fetal bovine serum (FBS) (Hyclone), 100 U/mL streptomycin and 100 μg/mL penicillin (Invitrogen, Carlsbad, CA, USA) in a humidified incubator at 37 °C with 5% CO_2_. Cycloheximide (CHX) (Sigma-Aldrich, St Louis, MO, USA) and MG132 (Selleck Chemicals, USA) were used at the indicated concentrations.

### Plasmids, siRNAs and cell transfection

The wild-type Flag-SP1(WT) or its mutants [Flag-SP1 (K610R), Flag-SP1 (K624R), Flag-SP1 (K685R), Flag-SP1 (K693R) and Flag-SP1(ALL)] were inserted into the GV141 eukaryotic expression vector (Genechem, Shanghai, China) using XhoI/KpnI sites. Small interfering RNA used for TRIM25 gene knockdown was produced by Genomeditech (Shanghai, China). The sequences of siRNAs were as follows: si-TRIM25 1 (si-RNA 1), 5′-GAGUGAGAUCCAGACCUUGAA-3′ (forward) and 5′-UUCAAGGUCUGGAUCUCAACUC-3′ (reverse); si-TRIM25 2 (si-RNA 2), 5′-GAACUGAACCACAAGCUGAUA-3′ (forward) and 5′-UAUCAGCUUGUGGUUCAGUUC-3′ (reverse); si-TRIM25 3 (si-RNA 3), 5′-GUGCCCGAUUCCUCUUAGAGA-3′ (forward) and 5′-UCUCUAAGAGGAAUCGGGCAC-3′ (reverse). A scrambled siRNA was used as the negative control. Plasmids and siRNAs were transfected into cells using Lipofectamine 3000 (Invitrogen).

### Mouse models

All animal experiments were in accordance with the Jiangsu Provincial Guidelines for the use of experimental animals, and it was approved by the Animal Care and Use Committee of Nanjing Medical University. Briefly, BALB/c nude mice (female, 6 weeks) were obtained from NLARSH China (Shanghai, China). Animals were housed in plastic cages and kept in a temperature-, humidity-maintained, and light-controlled room (23 ± 1 °C; 50 ± 5% humidity; 12 h light/dark cycle starting at 7:00 am). They had ad libitum accesses to food and water.

For establishing xenograft mouse models, 5 × 10^6^/200 μL of BGC823 cells were subcutaneously injected into flank of BALB/c nude mice. When the tumor volume (V = 0.5 × length × width^2^) reached about 100 mm^3^, the mice were randomly divided into different groups and received the indicated treatments. General situations and activities of each mouse were daily recorded. Tumor sizes were recorded by caliper every 3 days for calculating tumor volumes. Body weight of the experimental mouse was also recorded every 3 days. The endpoint of the experimental therapy was determined until enough growth (up to 2000 mm^3^) of the xenograft tumor or intolerable side effects.

**Fig. 1:**
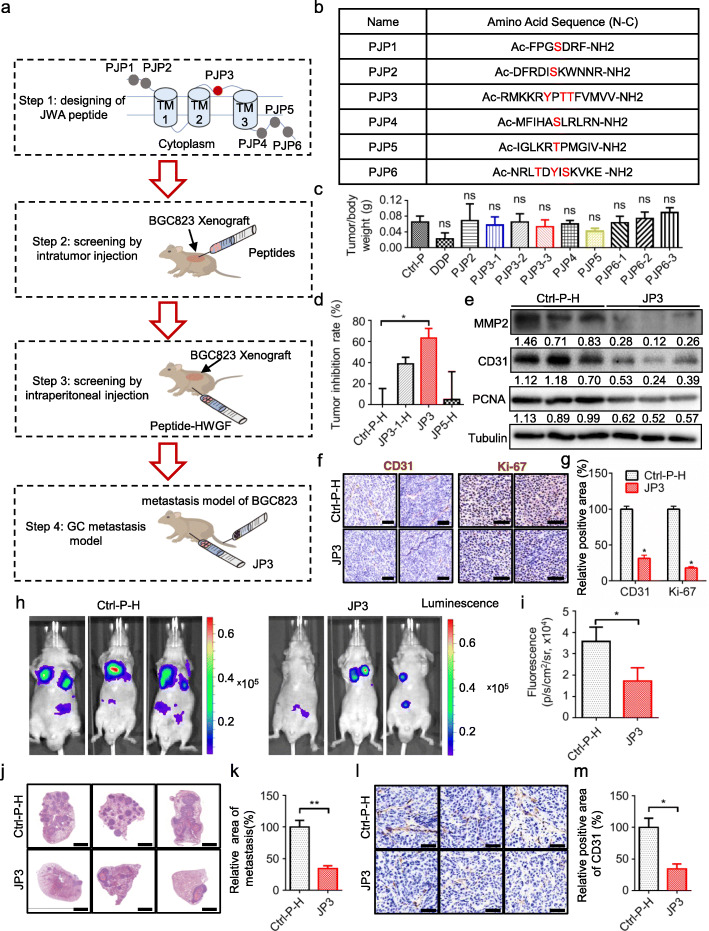
MMP2-targeted polypeptide JP3 inhibits growth and metastasis of GC in vivo. **a** The screening flowchart of JWA candidate peptides in nude mice. **b** The name and amino acid sequence of six fragments. **c** The ratios of mouse xenograft tumor weight/mice weight from screening model by intratumor injection. **d** The inhibition rates of tumor growth from screening model by intraperitoneal injection. **e** The expression levels of MMP2, CD31 and PCNA protein were analyzed using Western blotting in tumor tissues from nude mice bearing GC. **f-g** Sections of tumors were stained with anti-Ki-67 and anti-CD31 antibodies by IHC (scale bars = 50 μm). **h-i** The luciferase signals in the mice were detected and photographed using an IVIS in vivo image system. **j-k** The representative HE staining sections of lung (scale bars = 2 mm). **l-m** Sections of lung were stained with anti-CD31 antibodies by IHC (scale bars = 50 μm). The data are presented as the means ± SEM, ns: no significance, *P < 0.05, **P < 0.01

In the present study, the strategy to screen JWA protein based functional tumor inhibitory peptides included four steps, (1) all the candidate fragments were selected from non-transmembrane regions and contain amino acid can be phosphorylated in its sequence; (2) the intra-tumor injection in xenograft GC tumor in mouse model was used for first round screening for all candidate fragments; (3) the identified functional peptides in (2) were modified with HWGF as MMP2 target and used for secondary screening by intraperitoneal injection in xenograft GC mouse model; (4) the functional peptides selected in (3) were further used to inhibit metastasis of GC (Fig. 1a).

For establishment of metastatic GC mouse model, five-week-old BALB/c nude mice were randomly divided into two groups, 3 mice each. BGC823 cells were suspended in PBS. The mice were injected intravenously with 2 × 10^6^ cells in 0.15 mL of PBS through tail vein. On next day, the mice were treated by intra-peritoneal (ip) injection of Ctrl-H-P and JP3 and followed by luciferase substrate (GM-040611, Genomeditech, China); and photographed using the IVIS Spectrum in vivo imaging system (PerkinElmer, USA) on day 50^th^ to observe the cancer cell metastasis.

### Designing and synthesis of polypeptides

According to the predicted phosphorylation sites in 188 amino acids of JWA, six fragments (Fig. 1b) were selected and finally ten peptides were synthesized since only a single phosphorus modification to one amino acid residue was designed for each peptide. Both PJP3 and PJP6 fragments were split in three modified ones from each since three modifiable amino acid residues were contained in its sequences, respectively. A routine acetylation modification to each peptide was conducted to generate N-acetylation and C-amide, thereby increase its in vivo stability. According to the full length of JWA amino acid sequence and spatial structure, each synthesized peptide with a phosphorylated serine (S), threonine (T) or tyrosine (Y) in the center, and extension of 3–7 amino acid sequences in both sides. The MMP2-targeting motif HWGF was added after a four glycine (G) linker at C- termini of peptide (named as JP1-JP3).

All the peptides were synthesized by GL Biochem Ltd. (Shanghai, China), with a purity of more than 98%. These solid-phase synthesized peptides have good water solubility. The synthesized peptides were packed as 10 mg in sterile Eppendorf tube and always kept at − 20 °C. All peptides were dissolved in pre-cold PBS to the indicated doses/concentrations before use.

### Immunofluorescence assay

GC cells were treated with different concentrations of FITC-JP3 for different time points, followed by washing with phosphate buffer saline (PBS) for 3 times. Cells were then fixed with methanol for 15 min at room temperature and washed in PBST (PBS supplemented with 0.5% Tween-20). Cell nuclei were counterstained with DAPI (Beyotime, Jiangsu, China) for 20 min. The confocal images of stained cells were captured using the Zeiss AIM software on a Zeiss LSM 700 confocal microscope system (Carl Zeiss Jena, Oberkochen, Germany).

### Coimmunoprecipitation (co-IP)

BGC823 and SGC7901 GC cells were grown to confluence and processed for coimmunoprecipitation by standard procedures as previously described [16]. The pre-cleared lysate was incubated with anti-SP1 or anti-TRIM25 polyclonal antibodies for 1 h, and then incubated with protein A/G agarose beads overnight. Collected beads by centrifugation were washed for 5 times with washing buffer, and resuspended in 1× sodium lauryl sulfate loading buffer. Immunoprecipitation was eluted by incubation at 95 °C for 5 min.The eluted proteins were separated by sodium dodecyl sulfate - polyacrylamide gel electrophoresis, and then Western blotting with anti-SP1 (CST, Danvers MA, USA), anti-TRIM25 and anti-ubiquitin antibodies, respectively.

### Ubiquitination assay

GC cells were treated with or without JP3, followed by incubation with MG132 (10 μM) for another 6 h. Cells were harvested, prepared for protein samples and divided for Western blotting and co-IP assay, respectively. For co-IP assay, the protein (500 μg) was cultured with anti-SP1 or anti-TRIM25 antibody (dilution: 1:250) at 4 °C for 1 h, and Protein A/G Plus-Agarose overnight. After three cycles of washing in pre-cold IP buffer and centrifugation at 4 °C, 1000×g for 5 min, the immunoprecipitate was collected by centrifugation, eluted and examined by Western blotting.

### Western blotting analysis

Western blotting analysis was carried out as previously described. The antibodies against MMP2, Tubulin, Ub, HA-tag, Flag-tag and Ki-67 were purchased from Cell Signal Technology (Danvers MA, USA); anti-CD31 and PCNA were provided by Epitomics (MA, USA); anti-GAPDH and Actin were purchased from Beyotime (Jiangsu, China); anti-TRIM25 and SP1 were provided by Proteintech (Wuhan, China); anti-His was purchased from MBL (Japan). All above antibodies were used at a dilution of 1:1000.

### Quantitative real-time polymerase chain reaction (qRT-PCR)

According to the manufacturer’s protocol, total RNAs were isolated using TRIzol (Invitrogen, CA, USA) and reversely transcribed into cDNAs using reverse transcription kit and the SYBR Green Master Mix kit (Takara, Otsu, Japan). The complementary DNA (cDNA) was amplified with the following primers: 5′-GTCTCTACCCAGAACAGTTTCC-3′ (forward) and 5′-ATCCAACACAGGCTGATTCC-3′ (reverse) for TRIM25; 5′-GCCGGTGCTGAGTATGTC-3′ (forward) and 5′-CTTCTGGGTGGCAGTGAT-3′ (reverse) for GAPDH; 5′-GGTGCCTTTTCACAGGCTC’-3′ (forward) and 5′-GCTGTTCTCATTGGGTGACTC-3′ (reverse) for SP1. Quantitative RT-PCR was carried out an ABI Prism 7900 Sequence detection system (Applied Biosystems, Canada). Relative levels were normalized to that of GAPDH.

### HUVECs tube formation assay

After 6 hours of peptide treatment of GC cells, the medium was replaced and cultured in a 60 mm petri dish containing 1% serum for 24 h. On the next day, 2 mL of conditional medium was collected. For tube formation assay, a 96-well plate was coated with 50 μL of Matrigel™ (BD Biosciences) and kept at 37 °C for 2 h. Then, 1.2 × 10^4^ HUVECs suspended in 100 μL of conditional medium were applied to each well of the precoated 96-well plate. After incubation at 37 °C for 4 h, tubular structures formed in the Matrigel were captured in five randomly selected fields per sample under a microscope.

### Protein docking analysis

I-TASSER (Iterative Threading Assembly Refinement) was designed for protein structure modelling by iterative threading assembly simulations. The molecular structures of JP3, MMP2, and TRIM25 were constructed by I-TASSER. The largest possible binding pocket of MMP2 and TRIM25 was then predicted by Discovery Studio 3.0. These predicted pockets were utilized to construct an initial coarse model of the JP3-MMP2, JP3-MEK1/2 and JP3-TRIM25 complex. The coarse model of a complex was then refined using RosettaDock. Since a peptide was considered as a rigid body in RosettaDock, the peptide–protein complex with the lowest energy was then refined by utilizing the FelxPepDock module of Rosetta. The output of 2000 models was then ranked based on their energy scores. The model with the lowest energy was chosen for further research, i.e., binding sites and electrostatic properties. The binding sites between proteins were obtained based on space coordinates and types of amino acid (hydrogen bond: ≤3.5 Å; ionic: ≤3.5 Å; disulphide: ≤2.5 Å; van-der-waals: ≤0.5 Å; pi-pi interaction: ≤6.5 Å). Electrostatic interactions were found in almost all biomolecular systems and processes. To predict potential phosphorylation sites of TRIM25, a visual inspection of the electrostatic potential at a solvent-accessible surface was used. The electrostatic properties of the structures were calculated with the Adaptive Poisson–Boltzmann Solver (APBS) and PDB2PQR. High-quality 3D images of proteins were drawn with PyMOL/VMD.

### TMA construction and assessment of IHC

The construction of the GC TMAs was performed with standard procedures, and the assessment of the IHC employed a semiquantitative immunoreactivity score (IRS) as reported elsewhere [17]. The concordance for the IRS of the TRIM25 and SP1 staining scores between the two pathologists was 81 (90%) in 90 of the TMA cohort. The optimum value of cutoff points of the TRIM25 or SP1 IRS in this study were both 6 because the predictive value of this cutoff point for death was the best in the GC cohorts. Under these conditions, the samples with IRS scores 0–6 and IRS 8–12 were classified as low and high expressions of TRIM25 and SP1, respectively.

### Statistical analysis

All analyses and depicted graphs were performed with GraphPad Prism 6.0 Software (GraphPad Inc., San Diego, CA, USA). Data were expressed as mean ± SEM. The Student’s *t*-test was used to analyze differences between groups, while one-way ANOVA was used when more than two groups. In all statistical comparisons, *P value < 0.05 were considered as statistically significant (*P value < 0.05, **P < 0.01 and ***P < 0.001).

## Results

### JP3 suppresses GC angiogenesis, growth and metastasis in vivo

To identify tumor inhibitory functional fragment, we completed series screenings as described in Fig. 1a for all designed fragments (PJP1-PJP6) from JWA protein non-transmembrane regions (Fig. 1b); in fact, ten polypeptides were initially synthesized and used for screening since both PJP3 and PJP6 contained three residues could be phosphorylated, respectively. The first round screening of intra-tumor injection mouse models (10 mg/kg/d for 5 days/week× 2 weeks) showed PJP3–1, PJP3–3 and PJP5 (Additional files 1: Table S1) had better effects than the others (Fig. 1c); JP1 was previously ruled out because of its weaker tumor-suppressing effect on gastric cancer; as a positive control, although cisplatin showed better effects than all candidate peptides (Additional files 2: Figure. S1a), it was also indicated severe toxicity on mouse and with significant body weight lost since the 3^rd^ day of treatment (Additional files 2: Figure. S1b). The three candidates were further modified with four glycine and linked with HWGF for next screening by intraperitoneal injection (50 mg/kg/d for 12 days) in the GC cell xenograft mouse models. Gramm-x and Rosetta Dock simulation showed that 19His, 20Trp and 22Phe of the HWGF in JP3 could bind with MMP2 (Additional files 2: Figure. S1c). As shown in Fig. 1d, Additional files 2: Figure. S1d, JP3 indicated the best tumor inhibitory rate among candidates. We further confirmed whether the acetylation modification was necessary for JP3; as shown in Additional files 2: Fig. S1e-f, the inhibitory effect on GC xenograft was slightly better in acetylated JP3 group than in non-acetylated JP3 (Non-Ac-JP3) group. Western blotting showed JP3 treatment down-regulated expressions of MMP2, CD31 and PCNA significantly in GC tumor tissues (Fig. 1e); IHC data showed that JP3 suppressed expressions of CD31 (microvessel density (MVD)) and Ki-67 in GC tumor tissues (Fig. 1f, g). Colony formation assay showed no obvious effects of JP3 on GC cell proliferation in vitro (Additional files 3: Figure. S2). To determine whether JP3 inhibits GC metastasis, we completed a BGC823 GC cell metastatic mouse model. As shown by bioluminescence imaging, the fluorescence intensity of GC cell metastatic foci was obviously reduced in JP3 treated mice compared with the non-sense-HWGF peptide (Ctrl-P-H) group (Fig. 1h, i). HE staining showed that the relative area of metastatic tumor in lung from JP3-treated group was significantly decreased (Fig. 1j, k); IHC data showed that JP3 significantly suppressed expressions of CD31 in GC metastatic tissues (Fig. 1l, m). Collectively, these results suggested that JP3 suppressed GC tumor angiogenesis, proliferation and metastasis in vivo.

### JP3 inhibits GC cells mediated tubular formation of HUVECs

**Fig. 2:**
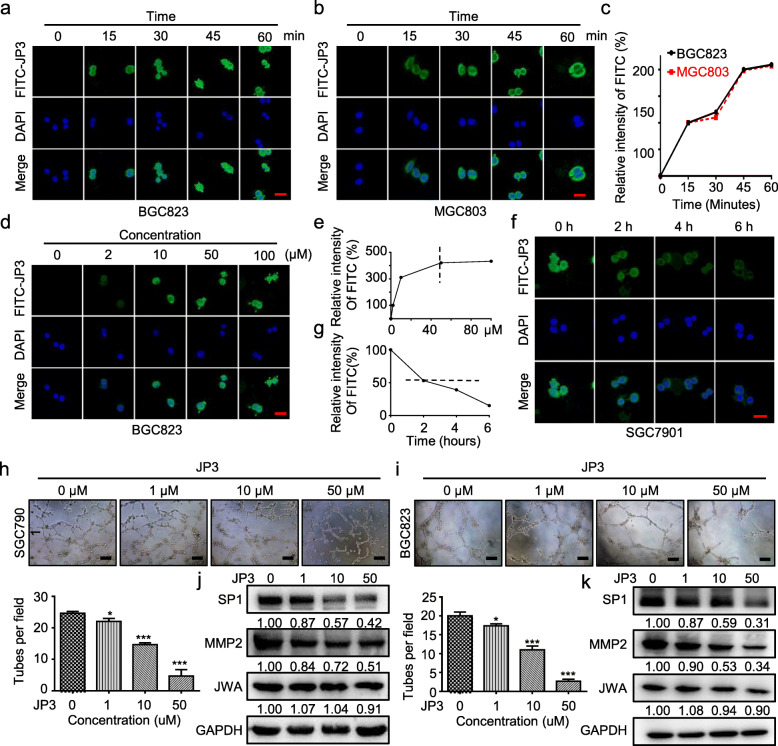
JP3 inhibits GC cells mediated tubular formation of HUVECs in vitro. **a-b** The subcellular localization and fluorescence intensity of BGC823 (**a**) and MGC803 (**b**) cells treated with FITC-JP3 for indicated time were determined by confocal laser scanning microscopy (scale bars = 50 μm). **c** The relative intensity of FITC fluorescence in MGC803 (red) and BGC823 (black) cells at different time points. **d** Representative images of SGC7901 cells treated with FITC-JP3 for 45 min (scale bars = 50 μm). **e** The relative intensity of FITC fluorescence in SGC7901 cells with different concentrations. **f** SGC7901 cells were treated with FITC-JP3 (50 μM) for 45 min, washed with PBS three times and cultured in normal medium. The cells were fixed for 0, 2, 4 and 6 h respectively (scale bars = 50 μm). **g** The half-life curve of FITC-JP3 was drawn. **h-i** Tube formation assay in HUVECs cultured with the medium collected from SGC7901 (**h**) and BGC823 (**i**) cells. Tubes were imaged (scale bars = 200 μm), and tube formation was quantified. **j-k** The indicated protein levels in SGC7901 (**j**) and BGC823 (**k**) were determined by Western blotting. The data are presented as the means ± SEM, ns: no significance, *P < 0.05, **P < 0.01

To determine how JP3 inhibits angiogenesis in GC, we first observed the pharmacokinetics of JP3 in GC cells. The FITC labelled JP3 (100 μM) was co-cultured with BGC823 (Fig. 2a) and MGC803 (Fig. 2b) GC cells for the indicated time; and JP3 peptide was rapidly entered cells and localized in cytoplasm; the intensity of fluorescence was reached a peak at about 45 min in both cells (Fig. 2c). JP3 was also showed a dose-dependent increase in GC cells and reached a peak at 50 μM (Fig. 2d, e); further observation showed that the intensity of JP3 in cells was decayed over time (Fig. 2f) and the intracellular half-life was about 2 h (Fig. 2g).

We then completed tubular formation assay in HUVECs using the conditional supernatent of JP3 treated GC cells. As shown in Fig. 2h, i, the supernatent from JP3 treated BGC823 and SGC7901 GC cells dose-dependently inhibited tubular formation. To determine whether JP3 also directly inhibited tubular formation, we treated HUVECs by JP3 at similar dose for 24 h, unfortunately no obvious effects were observed (Additional files 4: Figure. S3). Western blotting showed that JP3 treatment down-regulated expressions of SP1 and MMP2 but no obvious effect on JWA in GC cells (Fig. 2j, k). These results suggested that the effect of JP3 on tubular formation may be related to its inhibition of SP1 and MMP2 in GC cells therefore reduced MMP2 content in supernatants of culture medium.

### JP3 triggers ubiquitination modification of SP1 at K610 in GC cells

**Fig. 3:**
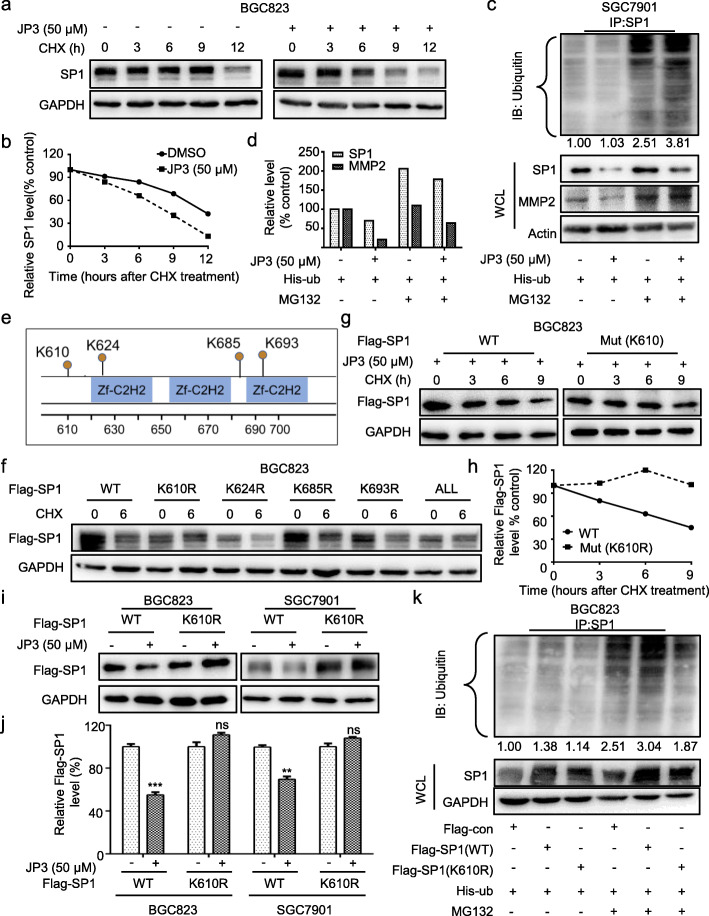
JP3 triggers ubiquitination modification of SP1 at K610 in GC cells. **a** BGC823 cells were treated with JP3 (0 or 50 μM), and then with CHX and harvested at the indicated time points for Western blotting. **b** The relative intensities of the SP1 protein bands were analyzed by densitometry after normalization to GAPDH. **c** Ubiquitination of SP1 was induced by JP3. His-ub was transfected into SGC7901 cells for 48 h and with JP3 (0 or 50 μM) for another 24 h, followed by pre-treatment with or without MG132 (10 μM) for 6 h. **d** The intensities of the SP1 and MMP2 protein bands in SGC7901 cells were analyzed by densitometry after normalization to Actin. **e** Data from the PhosphoSitePlus (https://www.phosphosite.org) showed the potential sites required for ubiquitination of SP1. **f** BGC823 cells were transfected with Flag-SP1 (WT) or mutants, followed by exposure to CHX (100 μg/ml) for 6 h. The indicated proteins were detected by Western blotting. **g-h** BGC823 cells were transfected with Flag-SP1 (WT) or Flag-SP1 (K610R) for 48 h and then JP3 (50 μM) for 24 h, followed by exposure to 100 μg/ml of CHX for 0, 3, 6, 9 h; the protein level of Flag-SP1 was determined by Western blotting, and the intensity of the SP1 protein bands were analyzed (**h**). **i, j** BGC823 (left) and SGC7901 (right) cells were transfected with Flag-SP1 (WT) or Flag-SP1 (K610R) for 48 h, followed by treatment with JP3 (50 μM) for 24 h, and **j** the intensity of the SP1 protein bands were analyzed. The data are presented as the means ± SEM, ns: no significance, **P < 0.01, ***P < 0.001. **k** BGC823 cells were co-transfected with His-Ub, Flag-SP1 (WT) or Flag-JWA (K610R) for 48 h, followed by pre-treatment with MG132 (10 μm) for 6 h. Ubiquitinated SP1 was determined by IP with anti-SP1. The indicated protein levels were determined by Western blotting

Our previous data have shown that JWA down-regulates SP1 expression by ubiquitination associated mechanism [12]. To confirm whether JP3 down-regulates SP1 through similar mechanism, we determined effects of JP3 on the stability of SP1 in GC cells. As shown in Fig. 3a-b, JP3 accelerated degradation of SP1 when BGC823 cells were treated with 50 μM JP3 for 24 h and then with CHX for indicated time. Ubiquitin modification assay showed ubiquitinated SP1 was increased in BGC23 (Additional files 5: Figure. S4a-b) and SGC7901 (Fig. 3c) cells after JP3 exposure; and MG132 treatment inhibited the downregulation of SP1 and MMP2 (Fig. 3c-d). However, the mRNA level of SP1 was not affected by JP3 treatment in both GC cells (Additional files 6: Figure. S5).

To determine the potential amino acids in SP1 protein that was ubiquitinated by JP3 in GC cells, we first conducted prediction assay via a web tool at https://www.phosphosite.org/. Data showed there were four potential lysine sites (K610, K624, K685 and K693) in the SP1 involved in JP3 induced ubiquitination. We then constructed Flag-tag SP1 mutants for these candidate amino acids and replaced lysine (K) with arginine (R), respectively (Fig. 3e). In addition, SP1 wild-type and four-K (ALL) mutant together with the four single K mutated plasmids were transfected into BGC823 cells separately for 48 h; and cells were treated with or without CHX for 6 h, respectively. Western blotting results showed that Flag-SP1 protein degradation did not occur in the cells transfected with SP1 (K610R) and SP1 (ALL mutant) plasmids compared with the other three mutants (K624R, K685R and K693R) transfected cells (Fig. 3f). The half-life of SP1 with K610R mutation was arrested compared with the wild-type SP1 (Fig. 3g, h) in cells. This was further confirmed in BGC823 and SGC7901 cells (Fig. 3i-j). Next, the ubiquitination of Flag-SP1 was examined, which was attenuated for the SP1 (K610R) mutant compared with that for SP1 (WT) (Fig. 3k). These results suggested that the K610 of SP1 was responsible for JP3 increased ubiquitination and degradation.

### TRIM25 interacts with SP1 and is negatively regulated by JP3 in GC cells

**Fig. 4:**
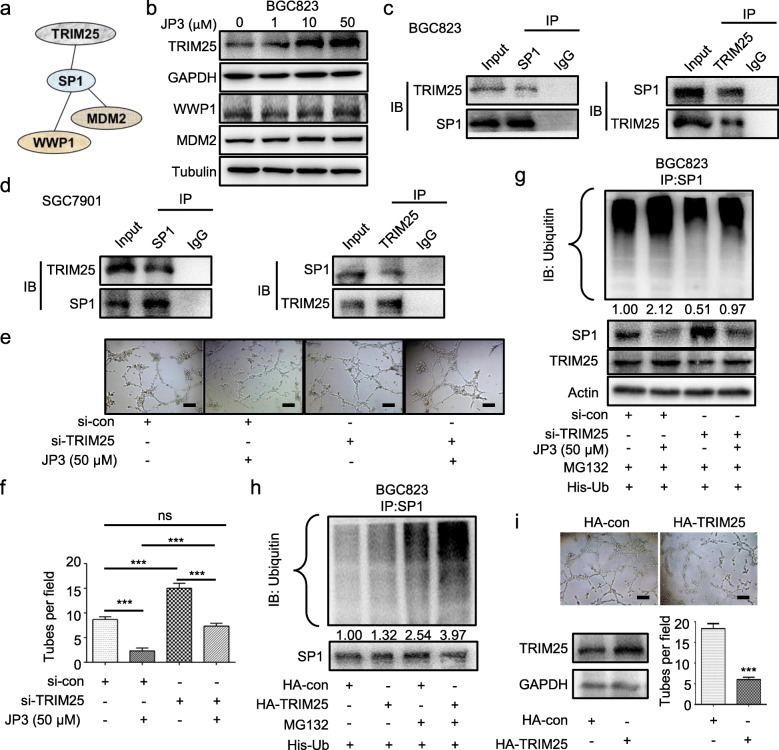
JP3 inhibits angiogenesis through degrading SP1 by E3 ubiquitin ligase TRIM25 in GC cells. **a** The relationship between the ubiquitin enzymes and SP1 was predicted online (http://genemania.org/). **b** BGC823 cells were treated with JP3 (0, 1, 10, 50 μM) for 24 h. The indicated protein levels were determined by Western blotting. **c-d** BGC823 (**c**) and SGC7901 (**d**) cells were pre-treated with MG132 (10 μM) for 6 h, and the endogenous protein-protein interaction between TRIM25 and SP1 was assessed by IP with anti-TRIM25 or anti-SP1 antibodies, followed by Western blotting. **e** si-TRIM25 was transfected into BGC823 cells for 48 h, followed by JP3 treatment for 24 h, and then, tube formation assay was performed. **f** The tube number was analyzed (means ± SEM, n = 3). ***P < 0.001. **g** BGC823 cells were co-transfected with His-Ub and si-TRIM25 or si-con for 48 h, and then treated with or without JP3 (50 μM) for another 24 h, followed by pre-treatment with MG132 (10 μM) for 12 h. Ubiquitinated SP1 was determined by IP with anti-SP1. The indicated protein levels were determined by Western blotting. **h-i** BGC823 cells were co-transfected with His-Ub and HA-TRIM25 or HA-con for 48 h, followed by pre-treatment with MG132 (10 μM) for 12 h, and then, tube formation assay was performed. Ubiquitinated SP1 was determined by IP with anti-SP1

To investigate how JP3 triggers ubiquitination at K610 of SP1, we completed prediction by online tools (http://ubibrowser.ncpsb.org/;http://genemania.org/). Among the top six of ubiquitin enzymes with the confidence scores higher than 0.7 (Additional files 7: Table S2), TRIM25, WWP1 and MDM2 may more reliable to interact with SP1 (Fig. 4a). We then determined their expressions in transcriptional and translational levels, respectively. As shown in Fig. 4b, only TRIM25 indicated a dose-dependent increase with JP3 treatment in BGC823 cells. The mRNA level of TRIM25 was not affected by JP3 treatment (Additional files 8: Figure. S6). Co-IP assay was performed and a physical interaction between TRIM25 and SP1 was identified in endogenous settings of BGC823 and SGC7901 cells (Fig. 4c-d).

To elucidate the functional roles of TRIM25 in the degradation of SP1 in GC, BGC823 cells were pre-transfected TRIM25 small interfering RNA for 48 h, and subsequently treated with or without JP3. Conditional supernatent was collected to culture HUVECs for additional 4 h. Data showed si-TRIM25 treated supernatent increased the number of tubes; in line with this, the expression of SP1 was increased by si-TRIM25, and JP3 could partly reverse the relationships between SP1 and TRIM25 (Fig. 4e-f). To confirm the regulations of JP3 on TRIM25 triggered SP1 degradation, we completed the ubiquitin modification assays in BGC823 cells. As shown in Fig. 4g, JP3 treatment enhanced ubiquitination of SP1 which was partially inhibited by si-TRIM25; in contrast, HA-TRIM25 increased ubiquitination in SP1 (Fig. 4h). The tubular formation assay further showed HA-TRIM25 suppressed tube numbers in HUVECs (Fig. 4i). These data suggested that JP3 promoted the degradation of SP1 by upregulation of TRIM25 in GC cells, further resulted in the reduction of tubular formation of HUVECs induced by supernatent of GC cells.

### JP3 enhances phosphorylation and stabilization of TRIM25 in GC cells

**Fig. 5:**
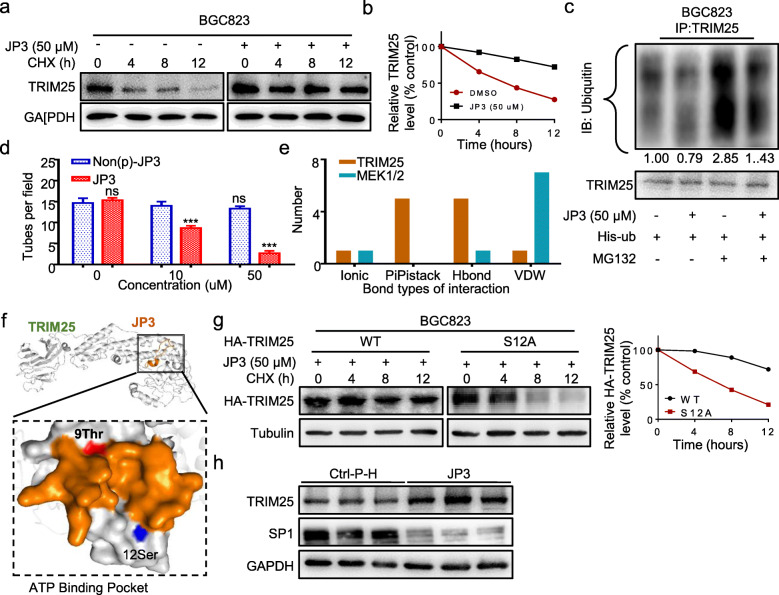
JP3 promotes the enhancement of phosphorylation at S12 and stabilization of TRIM25 in vitro. **a** BGC823 cells were treated with JP3 (0 or 50 μM), and then with CHX and harvested at the indicated time points for Western blotting. **b** The relative intensities of the TRIM25 protein bands were analyzed by densitometry after normalization to that of GAPDH. **c** BGC823 cells were transfected with His-Ub for 48 h, and then treated with JP3 (0 or 50 μM) for another 24 h, followed by pre-treatment with MG132 (10 μM) for 6 h. Ubiquitinated TRIM25 was determined by IP with anti-TRIM25, and the indicated protein levels were determined by Western blotting. **d** Tube formation assay was performed. The tube number was analyzed (means ± SEM, n = 3). ns: no significance, ***P < 0.001. **e** The main interaction types between amino acids between JP3 and TRIM25 or JP3 and MEK1/2. **f** A low-energy complex structure of JP3 and TRIM25 was predicted by a combination of Discovery Studio 3.0 and Rosetta software. **g** BGC823 cells were transfected with HA-TRIM25 wide type or S12A mutant for 36 h, followed by treatment of JP3 (50 μM) for another 12 h, treated with CHX and harvested at the indicated time points for Western blotting. The relative intensities of the HA-TRIM25 protein bands were analyzed by densitometry after normalization to that of Tubulin. **h** The expression levels of TRIM25 and SP1 protein were analyzed using Western blotting in tumor tissues from nude mice bearing GC

To investigate how JP3 modulates TRIM25, we determined effects of JP3 on the stability of TRIM25 in GC cells. As shown in Fig. 5a-b, JP3 slowed down the degradation of TRIM25 when BGC823 cells were treated with 50 μM JP3 for 12 h and then with CHX for indicated time. Ubiquitin modification assay showed ubiquitinated TRIM25 was decreased in BGC23 cells after JP3 exposure (Fig. 5c).

TRIM25 has been reported to auto-ubiquitylate itself. MAP3K13, a part of a non-canonical MAPK signaling pathway (PubMed: 28111074), phosphorylates TRIM25 at S12 and stabilizes it through decreasing its polyubiquitination [18]. As shown in Additional files 9: Figure. S7a, non-phosphorylated JP3 did not show obvious effects on SP1 and MMP2. Compared with JP3, the supernatant of Non(p)-JP3 treated GC cells was also no inhibitory role on tubular formation in HUVECs (Fig. 5d, Additional files 9: Figure. S7b). These results showed that the phosphorylation of JP3 was required for the inhibition of angiogenesis through increasing stability of TRIM25 by reducing its ubiquitination.

Our previous studies have proven that JWA affects tumor cells migration by activating the MAPK signaling pathway [19]. Given that TRIM25 was a specific E3 ubiquitin ligase on SP1 and was stabilized by JP3, we speculated that the activation of TRIM25 by JP3 may have two ways, one was MEK/MAPK, and the another one was a direct activation. JP3 binding capacity with MEK1/2 and TRIM25 were then analyzed based on predicted complex structures (Additional files 10: Figure. S8a, b). As shown in Fig. 5e, the main interaction types between amino acids of JP3 and TRIM25 were Hbond and PiPistack (Additional files 11: Table S3). However, VDW (van der Waals force) is the main interaction type between JP3 and MEK1/2. These results indicate that JP3-TRIM25 complex probably more stable than JP3-MEK1/2 complex. To determine whether JP3 directly interacts and activates TRIM25, we completed protein docking analysis using the PDB2PQR server. As shown in Fig. 5f, both the T9 site of JP3 and the S12 site of TRIM25 were distributed in the protein interaction region to form an ATP binding pocket, in which JP3 may transfer its phosphate group to and catalytic phosphorylation of S12 in TRIM25. This interaction was reversely confirmed by a non-phosphorylated JP3 (Non(p)-JP3). Data showed the non-phosphorylated T9 in JP3 had more positive potential and can’t bind with the S12 in TRIM25 (Additional files 10: Figure. S8c, Additional files 12: Table S4). We then constructed mutant and replaced S12 by alanine in TRIM25 to verify whether JP3 stabilizes TRIM25 protein expression by phosphorylating S12 sites. The TRIM25 wild-type and mutant plasmid were transfected into BGC823 cells for 48 h, followed by JP3 treatment for 12 h, with CHX for 0, 4, 8 or 12 h, respectively. The half-life of TRIM25 with S12A mutation was shortened compared with the wild-type TRIM25 (Fig. 5g). Finally, western blotting showed JP3 treatment up-regulated expression of TRIM25 and down-regulated expression of SP1 significantly in GC tumor tissues compared to the Ctrl-P-H treatment (Fig. 5h). Therefore, it was believed that JP3 could facilitate the interaction with TRIM25 and increased stability of TRIM25 by reducing polyubiquitination mediated degradation of itself. A stabilized and activated TRIM25 was necessary to down-regulate SP1 mediated expression of MMP2 in GC cells, and further inhibited tubular formation in HUVECs.

### Dysregulation of the TRIM25-SP1-MMP2 axis in GC

**Fig. 6:**
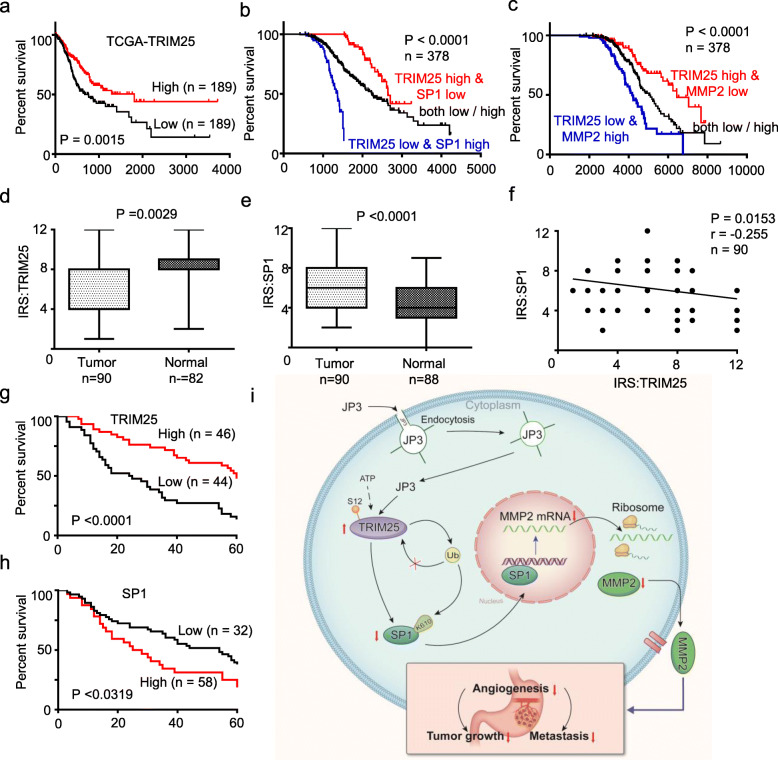
Dysregulation of the TRIM25-SP1-MMP2 axis in GC. **a-c** Kaplan–Meier Overall Survival curves of human GC patients with low versus high TRIM25 (**a**), combined with TRIM25/SP1 (**b**) and combined with TRIM25/MMP2 (**c**) expressions, based on TCGA data (http://www.oncolnc.org/). **d-e** Protein levels of TRIM25 and SP1 were detected in GC tumor tissues and normal tissues by IHC. **f** The correlations of the TRIM25 protein levels and SP1 protein levels were calculated (*n* = 90). **g-h** Kaplan-Meier curves depicting OS according to the expression patterns of TRIM25 (**g**) and SP1 (**h**) in the GC cohort. P values were calculated with the log-rank test. **i** A working model of JP3 on inhibiting tumor angiogenesis of CC via activating TRIM25 signaling pathway

To determine the significance of expressions both TRIM25 and SP1 in GC, using the online bioinformatics tool Kaplan-Meier Plotter (http://www.oncolnc.org/), we found that patients with a low level of TRIM25 in GC tissues suffer a poor overall survival (OS) than a high level of TRIM25 ones (Fig. 6a). When combining TRIM25 and SP1 or MMP2 together as a new variable, patients with GC were divided into three subgroups: high level of TRIM25 and low level of SP1 or MMP2; low level of TRIM25 and high level of SP1 or MMP2; and both low/high. Kaplan-Meier curves demonstrated that the subgroup of high level of TRIM25 and low level of SP1 or MMP2 was the most favorable to the overall survival (log-rank test, *P* < 0.0001; Fig. 6b-c). To confirm this, we completed assay on GC tissue microarray. Data showed that expression of TRIM25 was lower in GC tissues than in adjacent normal tissues (*P* = 0.0029, Fig. 6d); in contrast, expression of SP1 was higher in GC tissues than that in adjacent normal tissues (*P* < 0.0001, Fig. 6e); and the SP1 protein level was inversely correlated with the TRIM25 in primary tumors from GC patients (*r* = − 0.255, *P* = 0.0153, Fig. 6f, Additional files 13: Table S5). Further, the OS data showed the patients with low TRIM25 or high SP1 expression alone in GC tumor tissues had poor OS than high TRIM25 or low SP1 ones (Fig. 6g-h). Taken together, these data suggest that activation of the TRIM25-SP1 signaling could be benefit on OS in GC patients.

## Discussion

In this study, we identified a functional fragment from JWA protein and developed JP3 as a MMP2-targeted anti-GC peptide. We provided evidences at first time that JP3 suppressed xenograft tumor growth and metastasis in mouse models; the mechanistic data indicating that JP3 entered GC cells and activated TRIM25, and then increased degradation of SP1, therefore inhibited angiogenesis by reducing transcriptional activation on MMP2. JP3 increased SP1 degradation also by stabilizing TRIM25 through reducing its ubiquitination. It is also proven that K610 in SP1 was responsible for TRIM25 mediated ubiquitination. Collectively, we developed a MMP2-targeted peptide JP3 that exerts therapeutic roles on GC through inhibiting angiogenesis via the TRIM25-SP1-MMP2 signaling (Fig. 6i).

In recent years, the progress in the therapy of gastric cancer are very limit. The traditional first-line chemotherapy with platinum-based drugs is still the main regimen for gastric cancer [20, 21]. Although it is effective, its prominent disadvantages are severe side effects and drug resistance after repeated use [22, 23]. However, the emerging peptide drugs have the advantages of small molecular weight, strong tissue permeability, low immunity, high affinity with the target and few side effects [24, 25]. The designing strategy of JP3 was based on the anti-cancer functions and mechanisms identified in previous studies. The anticancer activity of JP3 was due to its 14 amino acid fragment sequence exactly come from JWA protein. Given previous studies indicated that the phosphorylation of JWA itself is necessary for its anti-cancer activity, whereas this JP3 fragment contains three amino acids that can be phosphorylated. By screening, we determined that JP3 is most effective only when its ninth amino acid T was phosphorylated. The designing of HWGF junctions targeting MMP2 was also suggested by previous studies. Here we demonstrated that JP3 plays a role in inhibiting angiogenesis, proliferation and metastasis of gastric cancer through down-regulating MMP2. It should be emphasized that HWGF targeting MMP2 alone has no biological function to inhibit gastric cancer. Our data also showed undetectable side effects in JP3 treated xenograft and metastatic mice models.

MMP2 is an identified molecule closely related to invasion, angiogenesis and metastasis in many cancers including GC [26–29]. In recent years, MMP2-targeted drugs have been well concerned, for example, both Marimastat and Tanomastat are MMP2-targeted drugs. Unfortunately, the both are eventually halted because of poor clinical outcomes [30]. Of note, JP3 increased stability and reduced ubiquitination of TRIM25 by de novo phosphorylation at Ser12. Upregulation of TRIM25, an E3 ubiquitin ligase, could increase the ubiquitination degradation of the transcription factor SP1 of MMP2, thereby reduced the transcription and translation of MMP2.

In the present study, we focused on the discovery of anticancer peptide JP3 and its mechanism of action. The limitations include the universality of JP3 in other tumors, since SP1 and MMP2 overexpressions exist in most malignancies [31]. In addition, we have demonstrated that JP3 can activate and stabilize TRIM25 through direct interaction. We need to further clarify whether JP3 can influence downstream signaling of TRIM25 through phosphorylation and ubiquitination by interacting with MEK/MAPK. In addition, the pharmacodynamics of JP3 as a potential anticancer polypeptide remain largely unknown, such as the distribution in other tissues and organs, the half-life in the peripheral blood. Finally, whether JP3 is suitable for tumor heterogeneity, and can be used in combination with other chemotherapy drugs like platinums are needed further investigation.

## Conclusion

In summary, we identified a novel, non-toxic therapeutic anti-angiogenic peptide JP3 for GC. Functional experiments demonstrated that JP3 suppresses GC cell growth and metastasis through TRIM25-SP1-MMP2 signaling axis.

## Supplementary information

**Additional files 1: Table S1.** The name and amino acid sequence of different peptides.

**Additional files 2: Figure S1.** The screening of JWA candidate peptides in nude mice. (**a**) Collected tumors in nude mice bearing GC after different peptides treatment (50 mg/kg, 5 d/w) were captured. **(b)** The curve of mouse body weight. **(c)** Computer simulation of JP3 and MMP2 binding sites. **(d)** Collected tumors in nude mice bearing GC after different MMP2-targeted polypeptides treatment were captured. **(e)** Ctrl-H, JP3 or Non-Ac-JP3 was respectively administered by daily intraperitoneal injection at a dose of 50 mg/kg/d, for about 12 days. At the end of the trail period, mice were sacrificed for collecting tumors and photographed. **(f)** The tumor inhibition ratio of mouse xenograft tumor weight.

**Additional files 3: Figure S2.** JP3 has no direct effects on GC cell proliferation in vitro*.* The colony numbers of BGC823 cells treated with different concentrations of JP3.

**Additional files 4: Figure S3.** JP3 has no obvious directly effects on HUVECs. Treated HUVECs by JP3 directly for 24 h, tubular formation assay was performed.

**Additional files 5: Figure S4.** JP3 promotes SP1 ubiquitination for degradation in BGC823 GC cells. (**a**) Ubiquitination of SP1 was induced by JP3. His-ub was transfected into BGC823 cells for 48 h with JP3 (0 or 50 μM) for another 24 h, followed by pre-treatment with or without MG132 (10 μM) for 6 h. Ubiquitination of the SP1 protein was immunoprecipitated using an anti-SP1 antibody and further detected the ubiquitin antibody. In whole lysates, endogenous SP1 and MMP2 were examined by the indicated antibodies. (**b**) The intensities of the SP1 and MMP2 protein bands in BGC823 cells were analyzed by densitometry after normalization to that of Actin.

**Additional files 6: Figure S5.** The mRNA level of SP1 is not affected by JP3 treatment in BGC823 and SGC7901.

**Additional files 7: Table S2.** The more reliable ubiquitin enzymes of SP1 predicted online (http://ubibrowser.ncpsb.org/).

**Additional files 8: Figure: S6.** The mRNA level of TRIM25 is not affected by JP3 treatment.

**Additional files 9: Figure S7.** Non(p)-JP3 does not show obvious inhibiting effect on angiogenesis. (**a**) BGC823 cells were treated with J Non(p)-JP3 for 24 h, and the indicated protein levels were determined by Western blotting. (**b**) Tube formation assay in HUVECs cultured with the medium collected from Non(p)-JP3 treated BGC823 cells.

**Additional files 10: Figure S8.** Model structure showing the interactions stabilizing JP3 and TRIM25 complexes. (**a-b**) JP3 binding capacity with MEK1/2 (**a**) and TRIM25 (**b**) were analyzed based on predicted complex structures. (**c**) The three-dimensional structures of Non(p)-JP3 and TRIM25 were predicted by I-TASSER (Iterative Threading Assembly Refinement) algorithm. The electrostatic properties of structures were then calculated using the PDB2PQR server.

**Additional files 11: Table S3.** The main interaction types between amino acids between JP3 and TRIM25.

**Additional files 12: Table S4.** The non-phosphorylated T9 in JP3 and S12 in TRIM25 have more positive potential and can’t bind with the S12 in TRIM25.

**Additional files 13: Table S5.** The numbers of cases among the 90 GC patients with the same IRS in TRIM25 and SP1.

## Data Availability

All other data are available in the main text or the supplementary materials. The datasets used and/or analyzed during the current study are available from the websites mentioned in the text.

## Abbreviations

GC: Gastric cancer
IHC: Immunohistochemistry;
TMA: Tissue microarrays
qRT-PCR: Real-time quantitative polymerase chain reaction
Co-IP: Coimmunoprecipitation
CHX: Cycloheximide
VDW: Van der Waals force
OS: Overall survival
MMP2: Matrix metalloproteinases 2
