# Supplementary material for: JP3, an antiangiogenic peptide, inhibits growth and metastasis of gastric cancer through TRIM25/SP1/MMP2 axis
Source: J Exp Clin Cancer Res. 2020 Jun 23;39:118. doi: 10.1186/s13046-020-01617-8 (PMC7310436; doi:10.1186/s13046-020-01617-8)
Supplement: Supplementary file 1 — Additional files 1: Table S1. The name and amino acid sequence of different peptides. [file 13046_2020_1617_MOESM1_ESM.pdf]

Name	Amino Acid Sequence (N-C)
Ctrl-P	Ac-EEMQRR-NH2
JP3-1	Ac-RMKKRY(-p)PTTFVMVV-NH2
JP3-3	Ac-RMKKRYPTT(-p)FVMVV-NH2
JP5	Ac-IGLKRT(-p)PMGIV-NH2
