# Supplementary figures and images for: JP3, an antiangiogenic peptide, inhibits growth and metastasis of gastric cancer through TRIM25/SP1/MMP2 axis
Source: J Exp Clin Cancer Res. 2020 Jun 23;39:118. doi: 10.1186/s13046-020-01617-8 (PMC7310436; doi:10.1186/s13046-020-01617-8)

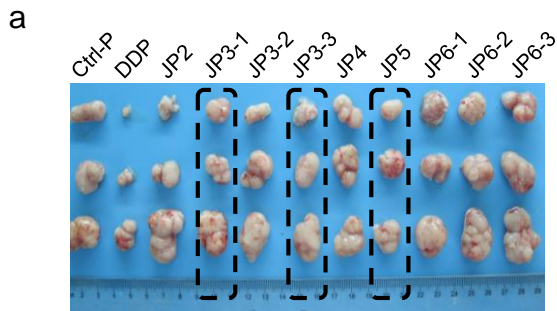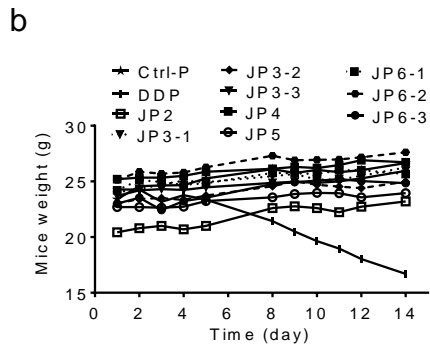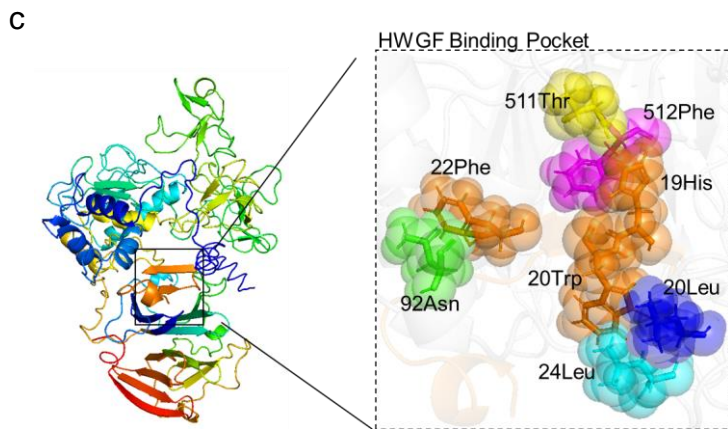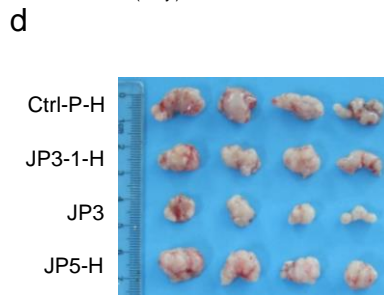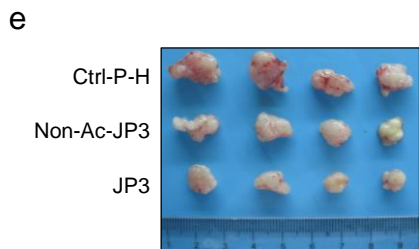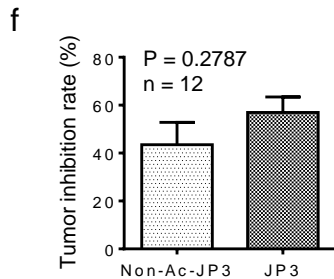

Supplement: Supplementary file 2 — Additional files 2: Figure S1. The screening of JWA candidate peptides in nude mice. (a) Collected tumors in nude mice bearing GC after different peptides treatment (50 mg/kg, 5 d/w) were captured. (b) The curve of mouse body weight. (c) Computer simulation of JP3 and MMP2 binding sites. (d) Collected tumors in nude mice bearing GC after different MMP2-targeted polypeptides treatment were captured. (e) Ctrl-H, JP3 or Non-Ac-JP3 was respectively administered by daily intraperitoneal injection at a dose of 50 mg/kg/d, for about 12 days. At the end of the trail period, mice were sacrificed for collecting tumors and photographed. (f) The tumor inhibition ratio of mouse xenograft tumor weight. [file 13046_2020_1617_MOESM2_ESM.pdf]

BGC823

JP3

0  $\mu\text{M}$

10  $\mu\text{M}$

50  $\mu\text{M}$

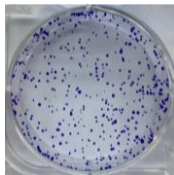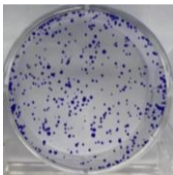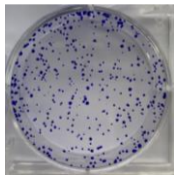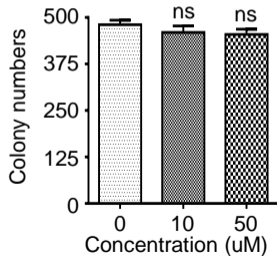

Supplement: Supplementary file 3 — Additional files 3: Figure S2. JP3 has no direct effects on GC cell proliferation in vitro. The colony numbers of BGC823 cells treated with different concentrations of JP3. [file 13046_2020_1617_MOESM3_ESM.pdf]

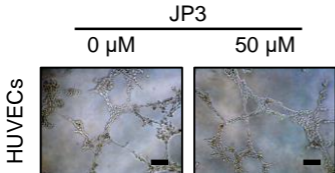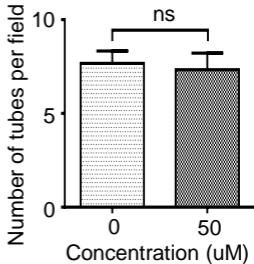

Supplement: Supplementary file 4 — Additional files 4: Figure S3. JP3 has no obvious directly effects on HUVECs. Treated HUVECs by JP3 directly for 24 h, tubular formation assay was performed. [file 13046_2020_1617_MOESM4_ESM.pdf]

a

BGC823  
IP:SP1

IB Ubiquitin:

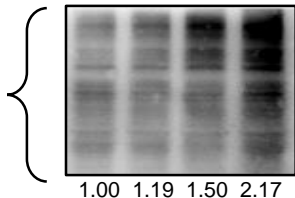

WCL

SP1

MMP2

Actin

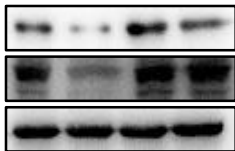

|                  |   |   |   |   |
|------------------|---|---|---|---|
| JP3 (50 $\mu$ M) | - | + | - | + |
| His-ub           | + | + | + | + |
| MG132            | - | - | + | + |

b

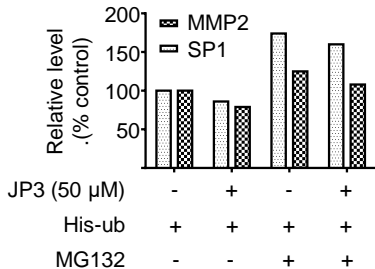

Supplement: Supplementary file 5 — Additional files 5: Figure S4. JP3 promotes SP1 ubiquitination for degradation in BGC823 GC cells. (a) Ubiquitination of SP1 was induced by JP3. His-ub was transfected into BGC823 cells for 48 h with JP3 (0 or 50 μM) for another 24 h, followed by pre-treatment with or without MG132 (10 μM) for 6 h. Ubiquitination of the SP1 protein was immunoprecipitated using an anti-SP1 antibody and further detected the ubiquitin antibody. In whole lysates, endogenous SP1 and MMP2 were examined by the indicated antibodies. (b) The intensities of the SP1 and MMP2 protein bands in BGC823 cells were analyzed by densitometry after normalization to that of Actin. [file 13046_2020_1617_MOESM5_ESM.pdf]

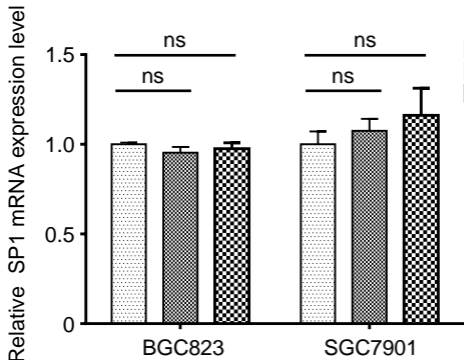

Supplement: Supplementary file 6 — Additional files 6: Figure S5. The mRNA level of SP1 is not affected by JP3 treatment in BGC823 and SGC7901. [file 13046_2020_1617_MOESM6_ESM.pdf]

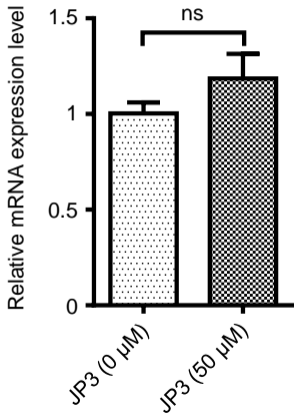

Supplement: Supplementary file 8 — Additional files 8: Figure: S6. The mRNA level of TRIM25 is not affected by JP3 treatment. [file 13046_2020_1617_MOESM8_ESM.pdf]

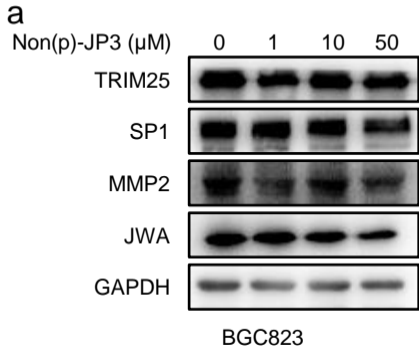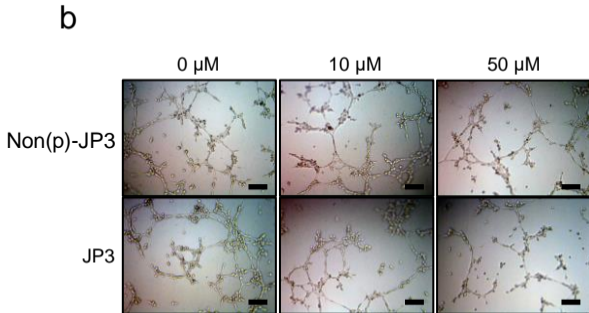

Supplement: Supplementary file 9 — Additional files 9: Figure S7. Non(p)-JP3 does not show obvious inhibiting effect on angiogenesis. (a) BGC823 cells were treated with J Non(p)-JP3 for 24 h, and the indicated protein levels were determined by Western blotting. (b) Tube formation assay in HUVECs cultured with the medium collected from Non(p)-JP3 treated BGC823 cells. [file 13046_2020_1617_MOESM9_ESM.pdf]

a

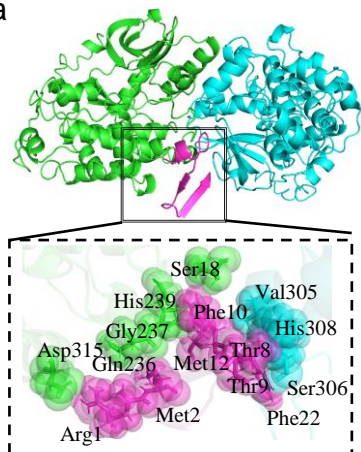

Chain: X (magenta) JP3  
 A (Green) : MEK1  
 B (Cyan) : MEK2

b

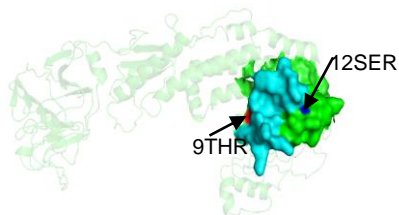

Chain: X (Green) TRIM25  
 A (Cyan) JP3

c

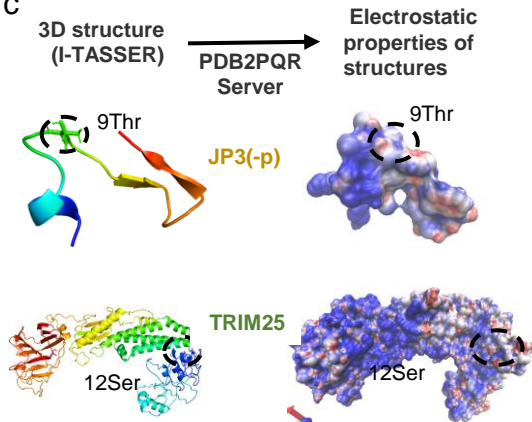

Supplement: Supplementary file 10 — Additional files 10: Figure S8. Model structure showing the interactions stabilizing JP3 and TRIM25 complexes. (a-b) JP3 binding capacity with MEK1/2 (a) and TRIM25 (b) were analyzed based on predicted complex structures. (c) The three-dimensional structures of Non(p)-JP3 and TRIM25 were predicted by I-TASSER (Iterative Threading Assembly Refinement) algorithm. The electrostatic properties of structures were then calculated using the PDB2PQR server. [file 13046_2020_1617_MOESM10_ESM.pdf]
