# Supplementary material for: JP3, an antiangiogenic peptide, inhibits growth and metastasis of gastric cancer through TRIM25/SP1/MMP2 axis
Source: J Exp Clin Cancer Res. 2020 Jun 23;39:118. doi: 10.1186/s13046-020-01617-8 (PMC7310436; doi:10.1186/s13046-020-01617-8)
Supplement: Supplementary file 7 — Additional files 7: Table S2. The more reliable ubiquitin enzymes of SP1 predicted online (http://ubibrowser.ncpsb.org/). [file 13046_2020_1617_MOESM7_ESM.pdf]

NO.	Gene symbol	Score	Confidence level
1	MDM2	0.835	HIGH
2	NEDD4L	0.825	HIGH
3	NEDD4	0.744	MIDDLE
4	WWP1	0.726	MIDDLE
5	SMURF1	0.721	MIDDLE
6	TRIM25	0.714	MIDDLE
