# Supplementary material for: JP3, an antiangiogenic peptide, inhibits growth and metastasis of gastric cancer through TRIM25/SP1/MMP2 axis
Source: J Exp Clin Cancer Res. 2020 Jun 23;39:118. doi: 10.1186/s13046-020-01617-8 (PMC7310436; doi:10.1186/s13046-020-01617-8)
Supplement: Supplementary file 11 — Additional files 11: Table S3. The main interaction types between amino acids between JP3 and TRIM25. [file 13046_2020_1617_MOESM11_ESM.pdf]

	JP3-TRIM25	JP3-MEK1/2
Ionic	Arg5-Asp142	Arg1-Asp315
Pipistack	His19-His109; Trp20-His109; Trp20-Phe135; Phe10-His183; Phe22-His183	NA
Hbond	Phe22-Lys112; Trp20-Asp136; Tyr6-Phe140; Arg5-Asp142; His19-Gln58	Thr9-His308
VDW	Thr9-Leu180	Met2-Gln236; Phe10-Ser18; Phe10-Gly237; Met12-His239; Thr8-His308; Phe10-Val305; Phe22-Ser306
