# Supplementary material for: JP3, an antiangiogenic peptide, inhibits growth and metastasis of gastric cancer through TRIM25/SP1/MMP2 axis
Source: J Exp Clin Cancer Res. 2020 Jun 23;39:118. doi: 10.1186/s13046-020-01617-8 (PMC7310436; doi:10.1186/s13046-020-01617-8)
Supplement: Supplementary file 12 — Additional files 12: Table S4. The non-phosphorylated T9 in JP3 and S12 in TRIM25 have more positive potential and can’t bind with the S12 in TRIM25. [file 13046_2020_1617_MOESM12_ESM.pdf]

Name	Atoms	Amino acids	Electrostatic values
Non(p)-JP3	N	9Thr	-0.4
	CA	9Thr	0
	C	9Thr	0.55
	O	9Thr	-0.55
	CB	9Thr	0
	CG2	9Thr	0
	OG1	9Thr	-0.49
	H	9Thr	0.4
	HA	9Thr	0
	HG22	9Thr	0
	HG21	9Thr	0
	HB	9Thr	0
	HG23	9Thr	0
	HG1	9Thr	0.49
TRIM25	N	12Ser	-0.4
	CA	12Ser	0
	C	12Ser	0.55
	O	12Ser	-0.55
	H	12Ser	0.4
	HA	12Ser	0
	CB	12Ser	0
	HB3	12Ser	0
	HB2	12Ser	0
	OG	12Ser	-0.49
	HG	12Ser	0.49
