# Supplementary material for: JP3, an antiangiogenic peptide, inhibits growth and metastasis of gastric cancer through TRIM25/SP1/MMP2 axis
Source: J Exp Clin Cancer Res. 2020 Jun 23;39:118. doi: 10.1186/s13046-020-01617-8 (PMC7310436; doi:10.1186/s13046-020-01617-8)
Supplement: Supplementary file 13 — Additional files 13: Table S5. The numbers of cases among the 90 GC patients with the same IRS in TRIM25 and SP1. [file 13046_2020_1617_MOESM13_ESM.pdf]

SP1 \ TRIM25	**1**	**2**	**3**	**4**	**6**	**8**	**9**	**12**
**1**								
**2**			1			1		1
**3**						1	1	1
**4**		1	2	3	2	5	4	4
**6**	1	2	2	7	2	10	5	2
**8**		3		3	8	6	3	
**9**				4	2	2		
**12**					1			
